# Comparing research investment to United Kingdom institutions and published outputs for tuberculosis, HIV and malaria: a systematic analysis across 1997–2013

**DOI:** 10.1186/s12961-015-0052-5

**Published:** 2015-11-04

**Authors:** Michael G. Head, Joseph R. Fitchett, Gemma Derrick, Fatima B. Wurie, Jonathan Meldrum, Nina Kumari, Benjamin Beattie, Christopher J. Counts, Rifat Atun

**Affiliations:** Farr Institute for Health Informatics, University College London, 222 Euston Road, London, NW1 2DA UK; Faculty of Medicine and the Global Health Research Institute, University of Southampton, Southampton, UK; Harvard T.H. Chan School of Public Health, Harvard University, Boston, USA; Health Economics Research Group, Brunel University, London, UK; Faculty of Medical Sciences, UCL Medical School, University College London, London, UK; Imperial College London, School of Medicine, London, UK; Johns Hopkins University School of Medicine, Baltimore, USA; London School of Hygiene & Tropical Medicine, London, UK; Northumbria Specialist Emergency Care Hospital, Northumbrian Rd, Cramlington, Northumberland UK

**Keywords:** AIDS, Bibliometrics, Funding, Health policy, HIV, Infectious disease, Malaria, Publications, Research impact, Research investments, Tuberculosis

## Abstract

**Background:**

The “Unfinished Agenda” of infectious diseases is of great importance to policymakers and research funding agencies that require ongoing research evidence on their effective management. Journal publications help effectively share and disseminate research results to inform policy and practice. We assess research investments to United Kingdom institutions in HIV, tuberculosis and malaria, and analyse these by numbers of publications and citations and by disease and type of science.

**Methods:**

Information on infection-related research investments awarded to United Kingdom institutions across 1997–2010 were sourced from funding agencies and individually categorised by disease and type of science. Publications were sourced from the Scopus database via keyword searches and filtered to include only publications relating to human disease and containing a United Kingdom-based first and/or last author. Data were matched by disease and type of science categories. Investment (United Kingdom pounds) and publications were compared to generate an ‘investment per publication’ metric; similarly, an ‘investment per citation’ metric was also developed as a measure of the usefulness of research.

**Results:**

Total research investment for all three diseases was £1.4 billion, and was greatest for HIV (£651.4 million), followed by malaria (£518.7 million) and tuberculosis (£239.1 million). There were 17,271 included publications, with 9,322 for HIV, 4,451 for malaria, and 3,498 for tuberculosis. HIV publications received the most citations (254,949), followed by malaria (148,559) and tuberculosis (100,244). According to UK pound per publication, tuberculosis (£50,691) appeared the most productive for investment, compared to HIV (£61,971) and malaria (£94,483). By type of science, public health research was most productive for HIV (£27,296) and tuberculosis (£22,273), while phase I–III trials were most productive for malaria (£60,491). According to UK pound per citation, tuberculosis (£1,797) was the most productive area for investment, compared to HIV (£2,265) and malaria (£2,834). Public health research was the most productive type of science for HIV (£2,265) and tuberculosis (£1,797), whereas phase I–III trials were most productive for malaria (£1,713).

**Conclusions:**

When comparing total publications and citations with research investment to United Kingdom institutions, tuberculosis research appears to perform best in terms of efficiency. There were more public health-related publications and citations for HIV and tuberculosis than other types of science. These findings demonstrate the diversity of research funding and outputs, and provide new evidence to inform research investment strategies for policymakers, funders, academic institutions, and healthcare organizations.

**Electronic supplementary material:**

The online version of this article (doi:10.1186/s12961-015-0052-5) contains supplementary material, which is available to authorized users.

## Background

The “Unfinished Agenda” of infectious diseases is of great importance to policymakers and funders of global health. The Global Burden of Disease study reaffirms the continuing high burden of communicable disease [1, 2]. The outbreak of Ebola in West Africa has illustrated the challenges faced by WHO and individual countries in effectively managing transmission across national borders and closing the gaps in global surveillance systems [3].

Peer-reviewed publications in academic journals – a typical output for funded research – can help to effectively disseminate the latest knowledge to policymakers, clinicians and other health professionals to inform policy and practice. Researchers at United Kingdom institutions have been prolific at publishing manuscripts in peer-reviewed journals [4]. Infectious disease is also a common topic of scientific papers [5, 6], and the vast majority of references within these papers are citing other journal articles [7]. Previous research analysing the returns from public research investment have concentrated on estimating the societal benefits received from research in the case of cancer [8]; cardiovascular and stroke research [9]; arthritis [10]; as well as from medical research in general [11]. However, this study is one of the first that directly links the publication return from public research investment. Although there have been criticisms of the emphasis applied to publishing in journals, and particularly in those with high impact factors [12], journals remain an important medium to sharing new knowledge and research findings. In addition, links between GDP and research productivity [13, 14] illustrate a policy need to understand a nation’s return on investment as an indicator of economic competitiveness and potential for growth.

The Research Investments in Global Health study [15] has systematically analysed investments between 1997 and 2013 in infectious disease research, highlighting funding levels by disease area [16], awarding body [17], receiving institution [18], and the sex of the principal investigator [19]. A 2015 publication showed that publication numbers for pneumonia broadly increased over time, with no clear relationship to changes in funding [20]. Herein, we consider three major infectious diseases, HIV, tuberculosis and malaria, and analyse individual trends in research investment and published outputs. We use a novel metric to assess the numbers of publications relative to research investments. We explore the impact of investment and published outputs by analysing citations for each disease area and by type of science.

## Methods

Awards for infectious disease research were sourced from the leading funders of infectious disease research in the public and philanthropic sectors. The compilation of the research investments data has been described in detail elsewhere [16, 20], but briefly reiterated here – we systematically examined investment data from 585 awarding bodies [21]. Data was obtained by searches on the funder website, requesting data directly from the funder, or searches on databases such as the National Research Register (now-archived and owned by the Department of Health) and ClinicalTrials.gov. Each downloaded study was examined for relevance to human infection. We excluded symposium grants, studies purely related to veterinary or plant infectious disease, and infrastructure grants unless there was clear emphasis on use for human infectious disease.

Studies from 1997 to 2010 were categorised under one of four types of science along the research pipeline – pre-clinical, phase I–III trials, product development, and public health research. In the updated analysis including the years 2011–2013, a fifth category, cross-disciplinary, was included in response to a perceived increase in awards that encouraged research across more than one type of science. Owing to resource constraints, this category has not yet been retrospectively applied to the 1997–2010 dataset. Awards were also categorised under a range of diseases and cross-cutting areas, including specific infections such as HIV, tuberculosis and malaria. Investment data across all years were adjusted for 2013 inflation, and awards in international currencies were converted to UK pounds using the average exchange rate in the year of the award.

Publications data from 1997–2013 was extracted from the Scopus database. Searches for article types were restricted to original article, editorial or review. Keywords searches were ‘AIDS’ OR ‘HIV’; ‘malaria’ OR ‘plasmodium’; and ‘tuberculosis’ OR ‘Mycobacterium’. By country, results were restricted to ‘UK’. All available information was downloaded into Excel spreadsheets, and conditional formatting equations applied in Excel to separate the list of authors into individual cells and thus be able to filter for publications with a United Kingdom-based first and/or last author. This criterion was used as a surrogate marker of significant United Kingdom involvement and thus presumed to be more comparable as a measure of outputs from United Kingdom research investments. Amongst the data available for analysis were publication title, abstract, article type, year of publication, journal title, and number of citations for each paper. Each publication was individually read by one of the authors and assessed for relevance to disease in humans for HIV/AIDS, malaria and tuberculosis, and grouped in one of the five types of science along the research and development pipeline used in the research investments categories (pre-clinical, phase I–III trials, product development, public health, cross-disciplinary).

To reduce inter-observer error, random samples of data were checked by a second author, with errors corrected and disagreements settled by consensus; a Cohen’s kappa score was calculated to measurement levels of agreement using GraphPad software [22].

In order to compare investments, publications and citations, a ‘UK pound per publication’ and ‘UK pound per citation’ metric was developed, across the three diseases and by type of science. The sum of investments across 1997–2010 was divided by the number of publications or citations from 1997–2013. Cross-disciplinary science was excluded from these analyses. Microsoft Excel 2013 and Stata V13 were used to assemble and analyse the datasets.

## Results

The number of publications extracted from Scopus was 19,461 for HIV, 9,355 for tuberculosis and 15,173 for malaria. Author categorisation produced 9,322 publications for HIV (47.9% of the initial number), 3,498 for tuberculosis (37.4%), and 15,173 for malaria (29.3%; Table 1). Major reasons for exclusion included keywords cross-cutting across different areas (e.g. ‘AIDS’ is also found in studies discussing ‘hearing aids’) and publications containing a United Kingdom author but not in first or last position. The agreement between authors (Cohen’s kappa) for categorisation was assessed as 0.88, rated as ‘very good’.

**Table 1:** **Summary of total research investment, publication and citation numbers for tuberculosis, HIV and malaria**

**Variable**	**HIV**	**Tuberculosis**	**Malaria**	**Total**
Investment 1997–2010, millions of UK pounds	515.7	168.0	381.5	1,065.2
Publications 1997–2013	9,322	3,498	4,451	17,271
Citations 1997–2013	254,949	100,244	148,559	503,752

**Figure 1:**
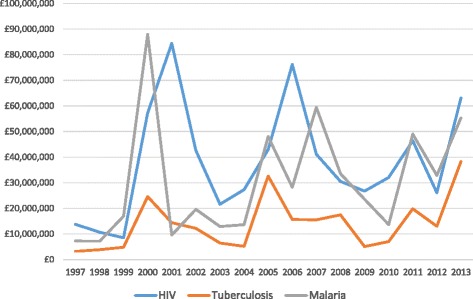
**Annual research investment for HIV, tuberculosis and malaria.**

Summary funding data have been published previously [16]. The total research investment (Table 1) for all three diseases was £1.4 billion and was greatest for HIV (£651.4 million), followed by malaria (£518.7 million) and tuberculosis (£239.1 million). Research investment per annum for each disease showed considerable variation (Figure 1). By type of science for each disease (Additional file 1), preclinical science received proportionately the greatest funding followed by public health and phase I–III, whereas product development awards received the least funding. A small amount of funding focused on cross-disciplinary studies across 2011–2013. Proportionate quantities for public health research increased for all infections in the later years of this dataset, typically at the expense of preclinical research. Similar findings were observed for publication and citation numbers (Additional file 1).

**Figure 2:**
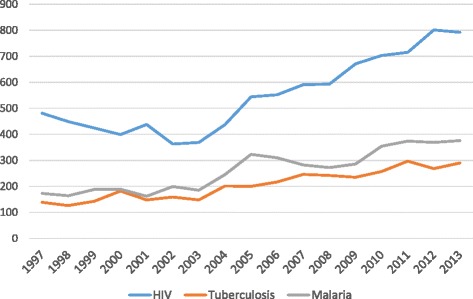
**Annual publication numbers for HIV, tuberculosis and malaria.**

There were 17,271 publications included for analysis (Table 1; 9,322 for HIV, 4,451 for malaria and 3,498 for tuberculosis). The predominant type of science (Additional file 1) was public health for HIV (62.9%) and tuberculosis (51.0%). Unlike research investments, publication numbers for all infections typically showed a steady increase year on year (Figure 2) from 793 in 1997 to 1,458 in 2013.

**Figure 3:**
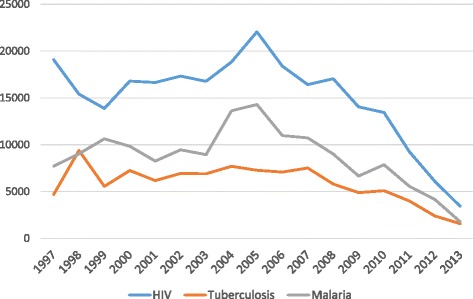
**Annual citations arising from publications relating to HIV, tuberculosis and malaria.**

The total number of citations (Table 1) was 503,752 and these showed more variability by year and a steady yearly decline for all three infections after 2006 (Figure 3). HIV publications received the most citations (254,949), followed by malaria (148,559) and tuberculosis (100,244; Table 1).

By investment per publication (Table 2), tuberculosis (£50,691) appears the most productive area for investment, compared to HIV and (£61,971) malaria (£94,483). By type of science (Additional file 1), public health was most productive for HIV (£27,296) and tuberculosis (£22,273), with phase I–III trials being the least productive (£326,440 and £187,185, respectively). For malaria, phase I–III trials were the most productive (£60,491) and all other types of science highlighted between £93 and £96,000 of investment per publication.

**Table 2:** **Relative investment in type of science for HIV, tuberculosis and malaria research, as described by a ‘UK pound per publication’ metric**

	**Preclinical**	**Phase I–III**	**Product development**	**Public health**	**Total**
	**HIV**				
Funding 1997–2010	£220,563,052	£111,316,004	£23,783,625	£160,119,168	£515,781,849
Publications 1997–2013	1,832	341	284	5,866	8,323
Pound per publication	£120,395	£326,440	£83,745	£27,296	£61,971
	**Tuberculosis**				
Funding 1997–2010	£96,477,706	£11,605,445	£20,172,857	£39,735,452	£167,991,460
Publications 1997–2013	1,275	62	193	1,784	3,314
Pound per publication	£75,669	£187,185	£104,523	£22,273	£50,691
	**Malaria**				
Funding 1997–2010	£202,459,316	£9,920,472	£12,839,850	£156,302,225	£381,521,863
Publications 1997–2013	2,112	164	138	1,624	4,038
Pound per publication	£95,861	£60,491	£93,042	£96,245	£94,483

By investment per citation (Table 3), tuberculosis (£1,797) appears the most productive area for investment, compared to HIV (£2,265) and malaria (£2,834). By type of science (Additional file 1), public health was the most productive for HIV (£2,265) and tuberculosis (£1,797), with phase I–III trials being the least productive (£7,479 and £4,035, respectively). For malaria, phase I–III trials were most productive (£1,713) and all other types of science highlighted between £2,600 and £3,200 of investment per publication.

**Table 3:** **Relative investment in type of science for HIV, tuberculosis and malaria research, as described by a ‘UK pound per citation’ metric**

	**Preclinical**	**Phase I–III**	**Product development**	**Public health**	**Total**
	**HIV**				
Funding 1997–2010	£220,563,052	£111,316,004	£23,783,625	£160,119,168	£515,781,849
Citations 1997–2013	80,109	14,883	8,677	124,020	227,689
Pound per publication	£2,753	£7,479	£2,741	£1,291	£2,265
	**Tuberculosis**				
Funding 1997–2010	£96,477,706	£11,605,445	£20,172,857	£39,735,452	£167,991,460
Citations 1997–2013	50,193	2,876	5,757	34,662	93,488
Pound per publication	£1,922	£4,035	£3,504	£1,146	£1,797
	**Malaria**				
Funding 1997–2010	£202,459,316	£9,920,472	£12,839,850	£156,302,225	£381,521,863
Citations 1997–2013	77,157	5,792	4,115	47,561	134,625
Pound per publication	£2,624	£1,713	£3,120	£3,286	£2,834

## Discussion

Across 1997–2013, significant public and philanthropic investments of over £1.4 billion have been awarded to United Kingdom institutions for HIV-, tuberculosis- and malaria-related research, with the vast majority of investment (£1.1 billion; 80.2% of total) directed to preclinical science or public health research. There were 17,271 published outputs on these disease areas, which were cited on 503,752 occasions. The most published and cited disease was HIV, and publications and citations were most numerous in preclinical science for HIV and tuberculosis, but for malaria this was phase I–III trials. The ‘investment per publication’ and ‘investment per citation’ metrics show tuberculosis to be the most productive area of research investment. By type of science, the metrics suggest public health research to be the most productive area for HIV and tuberculosis and phase I–III trials for malaria. The publication trends show that publication numbers steadily increase over time, distinct from the annual volatility of research funding.

HIV, tuberculosis and malaria are infections of huge global burden, and priority areas for WHO [23] and as well as the Global Fund, which has an annual budget of around US$4 billion and collaborates with local, national and international entities from the public, private and philanthropic sector with the aim of addressing and greatly reducing the impact of these diseases in the countries of highest burden [24]. Given the burden and international focus, the significant level of research investment is important and the extent of the publications and resultant citations unsurprising. However, funders and policymakers need to know how well their investments are performing and quantifications of the numbers and usefulness of the published outputs are an important measure of the impact and quality of research. The metrics developed herein highlight the more efficient performance of tuberculosis investments and (more generally) public health research, and will be of interest to the funding agencies and academic and clinical institutions which seek to engage in the highest-quality and best-performing research. It is also important that the large volume of knowledge generated by the research and disseminated by the publications is made clearly available to those involved in decision making regarding future funding priorities and more immediate considerations for policy and implementation.

The approach described herein covers three important disease areas, but it would be useful for future work to consider other infectious diseases and to systematically analyse investments and publications in non-communicable disease research. The investment dataset does not consider private sector investments, which may particularly impact upon the metrics used here for phase I–III trials and for product development research in these infections.

As journal requirements and publication databases evolve, it will become increasingly possible to link individual investment and published outputs via grant reference numbers and name of the agency sponsoring the research. This will provide the ability to generate more precise metrics of the relationship between investment and publications and citations. There is also the possibility for future work to consider published outputs by individual journal and journal metrics. Other research has suggested that the infectious disease content of the *Lancet* and *New England Journal of Medicine* journals consists of up to 35% for HIV and tuberculosis combined and 65% for all other infections [6]; therefore, the relative importance of each disease area for funders and publishers would be important to assess. Alongside the ResIn study [15], other research has highlighted the importance of the United Kingdom infectious disease research portfolio [25], and also the publication record of United Kingdom authors [4]. Funders such as the Wellcome Trust also use bibliometric analyses to assess their portfolio of studies [26]. Whilst detailed and informative, the disadvantage of these reports is that they focus solely on a single funder and each funder uses different criteria to analyse their work; one strength of this paper, and the ResIn study as a whole, is the unified approach across funders and disease areas, allowing simultaneous comparison of multiple awarding bodies.

The analysis in this study used data from one comprehensive publications database (Scopus) and inclusion of data from other databases may have modified the results as the content of each database is slightly different [ 27]. Categorisation of both investments and publications is necessarily a pragmatic process and open to subjectivity, though the rigour of the process is strengthened by the cross-checks of random samples of data by a second author. We made pragmatic decisions when applying our methodology – (1) that investments across 1997–2010 would publish most of their papers during 1997–2013; (2) that only including first or last authors from a United Kingdom institution would be a suitable measure of significant United Kingdom involvement and therefore likely to have received research funding. It is difficult to estimate how many papers have been excluded, or included, in error via these methods. Due to small numbers, we do not anticipate a significant impact of any retrospective reclassification of the cross-disciplinary category by type of science in the 1997–2010 data. Individual publications were assumed to be of equal impact and not controlled for by journal impact factor or any other publication or journal metric or weighting.

## Conclusions

The analyses reported herein suggest that tuberculosis is the best-performing disease area in terms of public and philanthropic research investment and publication and citation productivity, and public health is generally the type of science that is most prolific. These investments and publications generate great amounts of knowledge, and the analyses we report here can inform the thinking and priority-setting of policymakers such as WHO, national and international funding agencies, and the academic and clinical institutions which carry out research. The ResIn study [15] has secured funding from the Bill and Melinda Gates Foundation to carry out systematic analyses on investments and publications in infectious diseases in the G20 countries and so extended datasets will become available for open collaboration across 2016 and 2017.

## Additional file

Additional file 1:
**Supplementary information for publication.** (XLSX 49 kb)
